# How to Improve the Management of Acute Ischemic Stroke by Modern Technologies, Artificial Intelligence, and New Treatment Methods

**DOI:** 10.3390/life11060488

**Published:** 2021-05-27

**Authors:** Kamil Zeleňák, Antonín Krajina, Lukas Meyer, Jens Fiehler, ESMINT Artificial Intelligence and Robotics Ad hoc Committee, Daniel Behme, Deniz Bulja, Jildaz Caroff, Amar Ajay Chotai, Valerio Da Ros, Jean-Christophe Gentric, Jeremy Hofmeister, Omar Kass-Hout, Özcan Kocatürk, Jeremy Lynch, Ernesto Pearson, Ivan Vukasinovic

**Affiliations:** 1 Clinic of Radiology, Jessenius Faculty of Medicine in Martin, Comenius University in Bratislava, 03659 Martin, Slovakia; 2 ESMINT Artificial Intelligence and Robotics Ad hoc Committee, ESMINT, 8008 Zurich, Switzerland; office@esmint.eu (E.A.I.R.A.h.C.); daniel.behme@med.ovgu.de (D.B.); deniz.bulja@kcus.ba (D.B.); jildaz.caroff@gmail.com (J.C.); amar_chotai@yahoo.co.uk (A.A.C.); darosvalerio@gmail.com (V.D.R.); jcgentric.nri.brest@gmail.com (J.-C.G.); jeremy.hofmeister@hcuge.ch (J.H.); omarkasshout@gmail.com (O.K.-H.); ozcankocaturk@gmail.com (Ö.K.); jeremy.lynch@gmail.com (J.L.); ernestopearson@gmail.com (E.P.); vukasinovic_i@yahoo.co.uk (I.V.); 3 Department of Radiology, Charles University Faculty of Medicine and University Hospital, CZ-500 05 Hradec Králové, Czech Republic; antonin.krajina@fnhk.cz; 4 Diagnostic and Interventional Neuroradiology, University Medical Center Hamburg-Eppendorf, 20251 Hamburg, Germany; lu.meyer@uke.de (L.M.); fiehler@uke.uni-hamburg.de (J.F.); 5 University Clinic for Neuroradiology, Medical Faculty, Otto-von-Guericke University Magdeburg, 39120 Magdeburg, Germany; 6 Diagnostic-Interventional Radiology Department, Clinic of Radiology, Clinical Center of University of Sarajevo, 71000 Sarajevo, Bosnia and Herzegovina; 7 Department of Interventional Neuroradiology–NEURI Brain Vascular Center, Bicêtre Hospital, APHP, 94270 Paris, France; 8 Department of Neuroradiology, Royal Victoria Infirmary, Newcastle upon Tyne NE14LP, UK; 9 Department of Biomedicine and Prevention, University Hospital of Rome “Tor Vergata”, 00133 Rome, Italy; 10 Interventional Neuroradiology Unit, Hôpital de la Cavale Blanche, 29200 Brest, France; 11 Unité de Neuroradiologie Interventionnelle, Service de Neuroradiologie Diagnostique et Interventionnelle, 1205 Genève, Switzerland; 12 Stroke and Neuroendovascular Surgery, Rex Hospital, University of North Carolina, 4207 Lake Boone Trail, Suite 220, Raleigh, NC 27607, USA; 13 Balikesir Atatürk City Hospital, Gaziosmanpaşa Mahallesi 209., Sok. No: 26, 10100 Altıeylül/Balıkesir, Turkey; 14 Department of Neuroradiology, Toronto Western Hospital, Toronto, ON M5T 2S8, Canada; 15 CH Bergerac-Centre Hospitalier, Samuel Pozzi 9 Boulevard du Professeur Albert Calmette, 24100 Bergerac, France; 16 Department of Neuroradiology, University Clinical Center of Serbia, 11000 Belgrade, Serbia

**Keywords:** stroke, ischemia, ischemic stroke, management, diagnosis, treatment, artificial intelligence, rehabilitation, robotics, plan

## Abstract

Stroke remains one of the leading causes of death and disability in Europe. The European Stroke Action Plan (ESAP) defines four main targets for the years 2018 to 2030. The COVID-19 pandemic forced the use of innovative technologies and created pressure to improve internet networks. Moreover, 5G internet network will be helpful for the transfer and collecting of extremely big databases. Nowadays, the speed of internet connection is a limiting factor for robotic systems, which can be controlled and commanded potentially from various places in the world. Innovative technologies can be implemented for acute stroke patient management soon. Artificial intelligence (AI) and robotics are used increasingly often without the exception of medicine. Their implementation can be achieved in every level of stroke care. In this article, all steps of stroke health care processes are discussed in terms of how to improve them (including prehospital diagnosis, consultation, transfer of the patient, diagnosis, techniques of the treatment as well as rehabilitation and usage of AI). New ethical problems have also been discovered. Everything must be aligned to the concept of “time is brain”.

## 1. Introduction

In 2015, there were 6.3 million cerebrovascular disease deaths worldwide, making stroke the second leading global cause of death. Stroke deaths accounted for 11.8% of total deaths worldwide. A total of 3.0 million individuals died of ischemic stroke, and 3.3 million died of hemorrhagic stroke. Eastern Europe, East Asia, central Asia, and sub-Saharan Africa have higher rates of cerebrovascular disease mortality than the rest of the world. In 2010, there were an estimated 11.6 million events of incident ischemic stroke and 5.3 million events of incident hemorrhagic stroke, 63%, and 80%, respectively, in low- and middle-income countries [1,2]. Approximately 10% of all strokes occur in people 18 to 50 years of age [3].

Stroke is still one of the leading causes of death and disability in Europe. The European Stroke Action Plan (ESAP) for the years 2018 to 2030 defines four main targets: 1. to reduce the absolute number of strokes in Europe by 10%; 2. to treat 90% or more of all patients with stroke in Europe in a dedicated stroke unit as the first level of care; 3. to have national plans for stroke encompassing the entire chain of care from primary prevention to life after stroke; and 4. to implement national strategies for multisector public health interventions fully to promote and facilitate a healthy lifestyle and reduce the environmental (including air pollution), socioeconomic, and educational factors that increase the risk of stroke [4]. The cost of a one-minute treatment delay is tremendous. In each minute, 1.9 million neurons, 14 billion synapses, and 12 km (7.5 miles) of myelinated fibers are destroyed. Compared with the normal rate of neuron loss in brain aging, the ischemic brain ages 3.6 years each hour without treatment [5]. Everything must be aligned to the concept of “time is brain”. Several endovascular techniques were published with the aim of improving the recanalization rate and first-pass effect.

The COVID-19 pandemic forced the use of innovative technologies and created pressure to improve internet networks [6,7].

Robots for neurointerventional procedures are clearly on the rise. Diagnostic angiography, carotid artery stenting, and robot-assisted aneurysm coiling were performed successfully [8,9].

Cloud technologies provide real-time data analytics from various sources across integrated organizations and data sharing. This collaboration needs to be carefully balanced and information security must be guaranteed. Established large clouds might increase the risk of cyberattacks. Other problems include data ownership, patient monitoring, location identifiers, and disease classifications [10]. Despite security problems, massive data analysis has the potential to improve treatment decisions.

This review provides an introduction into possibilities of how to improve the management of acute ischemic stroke patients from the initial prehospital period to final rehabilitation and medical control by new technologies.

## 2. Stroke Management

To achieve the best results and reduce the morbidity and mortality of stroke patients, the management of stroke patients must be improved in all steps [11]. Utstein’s methodology for process improvement recommends the following strategies: 1. establish a stroke registry, 2. create public awareness, 3. start public education, 4. improve early recognition by first responders, 5. practice rapid and timely dispatch, 6. optimize prehospital stroke care and triage, 7. optimize in-hospital triage and acute care, 8. use smart technologies, 9. demonstrate accountability, and 10. create a culture of excellence [12].

Increasingly often, the implementation of modern technology makes patients more amenable to endovascular treatment. Prehospital diagnosis has enormous potential for improvement by the application of innovative technologies into daily life. Modifying stroke-screening tools is extremely important because it makes the treatment more accessible. The use of mobile stroke units, telestroke networks, mobile neuroendovascular teams, and smartphone applications has enormous potential to shorten the time window to treatment [13].

Artificial intelligence (AI) is used more and more often for radiologist education. AI-augmented radiology may enable precision medicine as well as precision medical education [14,15]. Robust data from stroke registries will be unbelievably valuable to find the right inclusion and exclusion treatment choice criteria.

The hierarchy of AI methods is as follows: 1. deep learning (DL) is a subset of 2. representation learning (RL), which is a subset of 3. machine learning (ML), which represents a subset of 4. AI [16]. Classic medical algorithms consist of three steps: 1. preprocessing of medical images, 2. Extraction, and 3. data classification, while modern algorithms do not request these steps due to neural networks [17]. Effectivity of various machine learning algorithms (e.g., linear regression, logistic regression, support vector machines, random forest, deep neural networks) are tested in medical studies.

Convolutional neural networks (CNNs) are increasingly often used for medical image classification [18]. Compared to acute stroke registry and analysis of Lausanne (ASTRAL), the deep neural network model was confirmed as the best form of tested machine learning algorithms to correctly predict outcomes in acute stroke patients, while random forest and logistic regression algorithms did not significantly differ (0.839 versus 0.888, 0.857, and 0.849) [19].

The deployment of AI represents a unique challenge for every nation and nowhere more so than in the improvement of health care [20]. In many European countries and Japan, an aging society and low AI development in health care ecosystems present an opportunity to improve in the field of novel AI technologies.

Supplementary Materials Table S1 is a summary about role of new technologies and artificial intelligence in acute stroke patient management.

### 2.1. Prehospital Diagnosis

A major limitation is that most of the general population is unable to recognize the symptoms of acute ischemic stroke correctly. Delayed first aid calls have a significant limiting effect on treatment outcomes. This lack is even more exacerbated in elderly people living alone. Therefore, this is an ideal point where modern technology can be implemented and doctors can obtain valuable time.

#### 2.1.1. Stroke Symptoms Recognition and First Aid Call

Different mobile-phone applications are available, but their quality and functionality differ according to several criteria: operating system, cost, target people, engagement, online interaction, release date, size, popularity, and usefulness. The information accountability of selected applications can be evaluated according to the Silberg scale. Although the applications targeting stroke patients at home have covered nine functionalities, most applications had only a simple functionality. The analysis confirmed that most applications did not offer a platform to facilitate online interaction with health professionals; however, this functionality is extremely crucial [21].

Independent systems of automatic location of the facial landmarks (i.e., points of lips, nose, eyes, and eyebrows) with a video-based approach for automatic orofacial assessment are promising to detect asymmetry instantly using 3D coordinates of facial points. Video-based technology is adding further evidence to the suitability for detecting orofacial impairments due to neurological diseases. The current accuracies obtained with this technique were up to 87%. Further clinical validation and correlation is the next step to daily routine usage [22].

#### 2.1.2. After First Aid Arrival

Technological aids to the diagnosis of large vessel occlusion or intracranial hemorrhage may contribute to uniform the use of clinical examination for stroke to facilitate efficient triage and improve transport decision making to endovascular-capable centers [23].

First, standardization of clinical examinations can be achieved by an application installed on smartphones (e.g., the FAST-ED), which can be reliable for paramedics with less training and occasional exposure to stroke, without comparable experience to highly trained hospital staff [24]. Such applications have the potential to improve triage, reduce hospital arrival times, and maximize treatment [24].

The next improvement can be achieved by automated scoring of patient facial palsy. The eFACE assessment obtained via photographs exhibits excellent inter-rater reliability and strong agreement with in-person assessment, demonstrating that facial symmetry in facial palsy patients can be monitored using standardized frontal photographs. Intraclass correlation coefficients for eFACE scores were 0.96 (95% CI 0.94 to 0.97) for total scores [25].

Intelligent decision-making algorithms systems lead to physicians’ empowerment, significantly reduces medical errors, and improves documentation quality. The recent study confirmed documentation quality (completeness) increased from 78.66% to 100% [26].

AI can improve confidence and reliability in nonexpert reading Mobile CT and prehospital transcranial Doppler results, technics routinely unused in prehospital triage nowadays, to improve decisions to start remote access to endovascular stroke experts, and thereby increases tPA administration rate and shortens time to tPA and endovascular treatment [27].

### 2.2. Team Activation, Consultation, and Transfer

The use of telemedicine/telestroke resources and systems should be supported by health care institutions, governments, payers, and vendors as one method to ensure adequate 24/7 coverage and care of acute stroke patients in a variety of settings. For sites without in-house imaging interpretation expertise, teleradiology systems are recommended [28]. A comprehensive telestroke network and interconnected system of endovascular centers must be established to create an effective health care system [29,30].

The ambulance needs real-time traffic information and the nearest stroke center capabilities [24]. The time for patient transport to the appropriate hospital is the primary criterion, the determined distance between the substantial position of the ambulance and the local hospital is less important. The functional system must naturally have up-to-date traffic information. It is extremely critical in high-density population areas such as metropolitan areas. Such a logistic system is currently used in Sydney for the selection of the nearest center according to arrival time, not according to the real distance between the GPS of the ambulance and the hospital [31].

Knowledge of the GPS location of the ambulance and estimated time for patient hospital arrival is also important for an endovascular center. The team is ready before the patient comes but all types of equipment can be used effectively, and the workflow is not blocked for a long time. Such a practical function is available on the STEMI application [32].

### 2.3. Prenotification—Stroke Team Activation before Patient Arrival

Patient information must be protected during communication between team members. Data protection must be guaranteed by the government or official representatives of the health care system. To facilitate communication and achieve appropriate patient treatment, nonofficial applications are used [33,34,35]. Such situations can potentially create data leaks. Although for the patient it’s probably more acceptable than a treatment delay, developing an AI-compatible international official stroke framework to protect personal information and enable responsible data sharing and cross-border data transfers in the European community would be beneficial to all [36].

The usefulness of using smartphone applications has recently been proven also in Egypt. The use of the Egyptian Stroke Network (ESN) application expedited the stroke treatment workflow and facilitated teleconnection between registered stroke facilities. The use of the application was associated with the significant drop in time metrics for the reperfusion AIS patients (door-in-door-out time; 56 ± 34 min vs. 96 ± 45 min, door-to-groin puncture time; 50 ± 7 min vs. 120 ± 25 min, door-to-needle time; 55 ± 12 min vs. 78 ± 16 min with *p* < 0.0001). Its use was also associated with higher rates of excellent outcomes at the 90-day follow-up (without ESN-app vs. with ESN-app, 67.9 vs. 47.1%, *p* = 0.001) [37].

### 2.4. Hospital Diagnosis

The statistical tool included in the app (e.g., “Stroke Clock” App) can be used to identify critical time-consuming processes within the stroke treatment workflow [38]. The mobile application can be comfortably installed on modern Android and iOS devices. With extensive knowledge, which is precisely the most time-consuming activity, hospital management can be focused on their continuous optimization. The exact time measurement is therefore beneficial.

Stroke clock display installed at CT room significantly reduced management metrics as treatment decision time (from 26.00 min to 16.73 min), end of neurological examination (from 10 min to 7.28 min), end of computed tomography examination (from 14.00 min to 11.17 min), and CTA (from 17.17 min to 14.00 min) [39].

Without access to the mobile CT unit, the final diagnosis must be confirmed in the hospital. Optimizing intrahospital management is extremely important. One-stop management, i.e., to use flat panel CT and CTA modality for the brain imaging (bypassing both the emergency department and multidetector computed tomography) has been suggested as an efficient method to reduce in-hospital delays [40,41].

Meta-analysis of data from seven randomized trials confirmed that the probability of reperfusion declined significantly with the time between hospital arrival and groin puncture. Among the 728 included patients, with a mean (SD) age of 65.4 (13.5) years old and of whom 345 were female (47.4%), decreases in rates of successful reperfusion, defined as thrombolysis in cerebral infarction score of 2b/3, were observed with increasing time from admission or first imaging to groin puncture. The magnitude of effect was a 22% relative reduction (odds ratio, 0.78; 95% CI, 0.64–0.95) per additional hour between admission and puncture and a 26% relative reduction (odds ratio, 0.74; 95% CI, 0.59–0.93) per additional hour between imaging and puncture [40].

Psychogios et al. reported study of 230 patients and a significant reduction of median door-to-reperfusion time (from 102 to 68 min; *p* < 0.001) and significantly better rate of good functional outcome (*p* = 0.029) in the one-stop management group [42]. Fewer radiation doses and fewer posterior fossa artifacts can be achieved by CBCT usage [42].

A broad range of AI benefits is expected to be delivered soon, including 1. lower radiation dose, 2. image quality improvement with fewer motion artifacts, 3. 40–60% faster scanning duration, and 4. positive impacts on workflow [43].

Even the standard usage of AI in modern medicine has great potential, and therefore, better evidence in the literature is necessary. Currently, only a limited number of prospective randomized trials focusing on AI in medical imaging have been published, usually with small human comparator groups and the possible risk of bias. Future studies should diminish the risk of bias, enhance real-world clinical relevance, instantly improve accurate reporting and transparency, and appropriately temper conclusions [44].

#### 2.4.1. Patient Registration

To avoid time lost, it is advisable to obtain the patient’s ID information before his/her arrival at the hospital. It allows us to start scanning at once after the placement of the patient in the CT or MR scanner. In such cases, we can skip the emergency scanning mode. Data can be easily merged with angiography.

Smartphone-based triage significantly reduces the wrong registration of patients in hospital from 0.68% (12,810/1,895,829) to 0.12% (2379/2,017,921) (*p* < 0.001) [45].

#### 2.4.2. Brain Imaging

Proper stroke management depends on specific information from imaging studies. Most steps in the triage naturally involve the presence of humans, and this is time limiting. The development of automated methods for stroke imaging evaluation is therefore required [18].

AI demonstrated remarkable progress in image recognition tasks. Historically, in radiology practice, trained physicians visually assessed medical images for the detection, characterization, and monitoring of diseases. AI methods excel at automatically recognizing complex patterns in imaging data and providing quantitative, rather than qualitative, assessments of radiographic characteristics [46].

The widespread use of AI in brain imaging is already known. At CT, patient positioning and examination planning contribute significantly to the total examination time; suboptimal imaging is a source of diagnostic errors and repeated examinations in any modality. Similarly, image reconstruction could undergo rapid adoption of AI algorithms to reduce the scan time, reduce contrast material doses, and increase image quality at MRI or for dose reduction at CT [47].

Over the past several years, a few software platforms have been commercialized of which some of the most popular are RapidAI (iSchema View, MenloPark, California, USA), e-Stroke Suite (Brainomix, Oxford, England) in collaboration with Olea Sphere (Olea Medical Solutions, La Ciotat, France), and Viz.ai (Viz.ai, SanFrancisco, California, USA) [17,48,49].

Developed software already allows several functionalities, including 1. the independent evaluation of brain involvement in terms of cerebral ischemia, 2. direct arterial occlusion detection, 3. collateral score quantification, 4. perfusion maps calculation, and 5. hemorrhage detection.

The ability to accurately predict the final volume of cerebral ischemia is in progress.

##### ASPECTS

Alberta Stroke Program Early CT Score (ASPECTS) is one of the most important criteria for the management of acute stroke patients. Those with proven blood vessel occlusion in the unknown and longer time windows may also be selected for mechanical thrombectomy based on ASPECTS and clinical criteria [50].

Interobserver agreement was evaluated in recent study. The ASPECTS region with highest level of agreement was the insular cortex (percent agreement = 96%, interrater agreement = 0.96 (95% CI: 0.94–0.97)), and with lowest level of agreement, the M3 region (percent agreement = 68%, interrater agreement = 0.39 [95% CI: 0.17–0.61]) [51]. Inappropriate scoring can naturally lead to wrong patient management.

To achieve appropriate interpretation of CT images, dedicated reconstruction algorithms are recommended. CT image postprocessing affects either automated or human ASPECTS in acute stroke patients. This effect was most pronounced by the less experienced readers, while the software had the most robust performance [52]. AI has the potential to play an important role in the standardization of ASPECTS evaluation and correct treatment indications.

**Figure 1:**
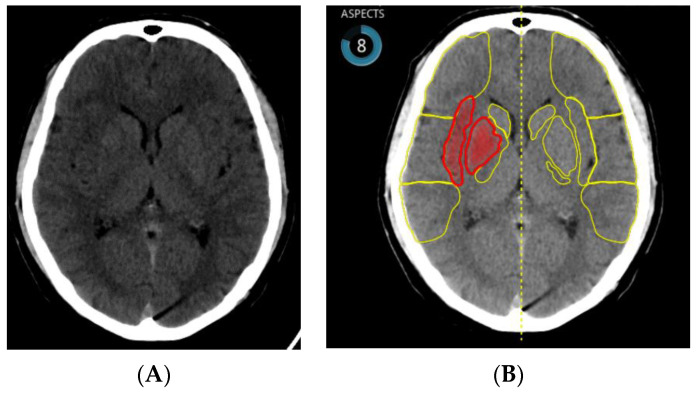
(**A**) NCCT brain scan—ischemic changes in territory of the right middle cerebral artery (insula and basal ganglia) and (**B**) identical patient—ischemia detected by artificial intelligence (ASPECT score = 8).

One of the early studies confirmed the noninferiority of e-ASPECT (Figure 1), compared to three neuroradiologists (*p* < 0.003) [53]. This is why nowadays AI can, apparently, offer the highest benefit in local hospitals with less extensive experience.

It is necessary to collect more information and conduct more studies to decide which software has the highest level of sensitivity and specificity. One study included 52 patients and 2 different software packages were tested. No clinically significant difference was observed for the total ASPECT score between human or automated packages, but there were differences in the characteristics of the regions scored. Package A was more sensitive in cortical areas than the other methods but at a higher level of specificity. Software package B had greater sensitivity but lower specificity for deep brain structures [54].

Variations of AI, including machine learning methods of random forest learning (RFL) and convolutional neural networks (CNNs), are used. ASPECTS commonly used RFL. A systematic literature review confirmed that image feature detection had greater sensitivity with CNN than with RFL, 85% versus 68% [55].

##### Artery Occlusion Detection

**Figure 2:**
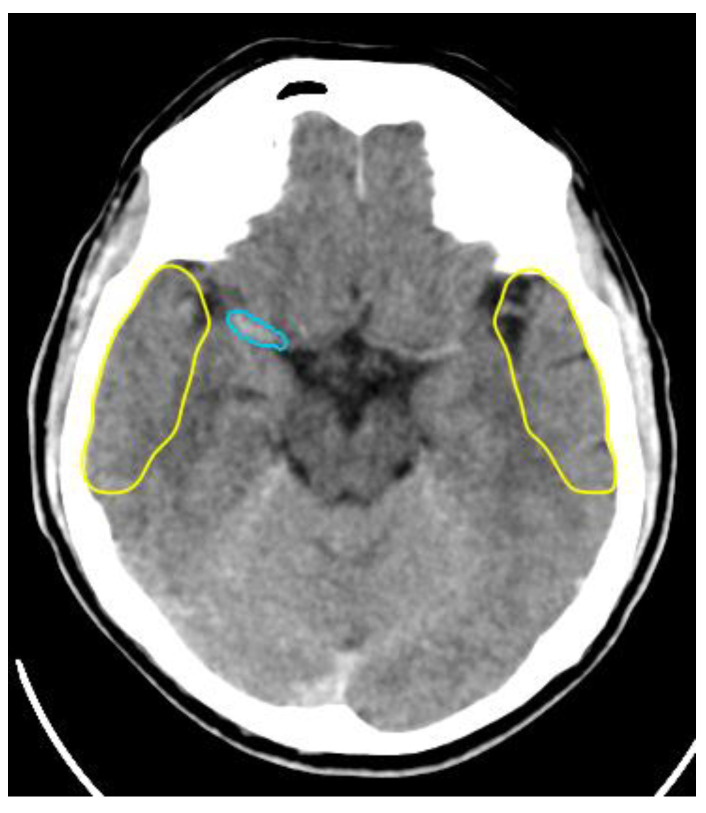
Detection of right middle cerebral artery occlusion by artificial intelligence from NCCT scan.

Large vessel occlusion (LVO) detection is essential for identifying potential candidates who could benefit from mechanical thrombectomy. CTA is typically the preferred technique for large vessel occlusion detection, but noncontrast-enhanced CT (NCCT) scans can also be used to localize the site of occlusion (Figure 2), and decrease the necessary amount of contrast media and reserve it only for angiography during recanalization procedure [16].

By the use of a support vector machine (SVM) for detection of the hyperdense middle cerebral artery (MCA) dot sign on NCCT, a maximum sensitivity of 97.5% (39/40) at a false-positive rate of 1.28 per image was achieved [56].

AI may improve LVO stroke detection and rapid triage necessary for expedited treatment. The AI algorithm of convolutional neural networks (CNNs) is typically used to detect LVO strokes [55].

Analysis of 650 CTAs by Viz LVO (Viz-AI-Algorithm^®^ v4.1.2.) demonstrated a sensitivity of 82%, specificity of 94%, PPV of 77%, and NPV of 95%. There were 31 false positives (16 due to intracranial atherosclerosis) and 23 false negatives. In total, 41 cases were not processed due to metal artifact, inadequate contrast, motion artifact, or excess z-spacing variability. The mean processing time for Viz.ai was 5 min with a maximum of 8 min for all studies. The mean clinical standard-of-care notification time at our institution was 32 min with a maximum of 116 min [57].

Similarly, when analyzing 1453 patients with suspected acute stroke, Olive-Gadea et al. found that a deep learning algorithm could even accurately identify large vessel occlusion on NCCTs, with an AUC of 0.87, and that this identification is improved (AUC of 0.91) when adding clinical parameters to AI system (such as NIHSS and time from symptom onset) [58].

The next challenge is the detection feature to identify distal vessel occlusion and even to suggest the histological thrombus composition [59].

##### Collateral Score

The collateral score may help to optimize the care of stroke patients. Patients with good collateral blood flow showed benefits, whereas those with poor collateral flow evidence showed less or no benefit. Time to reperfusion and collateral score are still main predictors of clinical outcomes [60].

**Figure 3:**
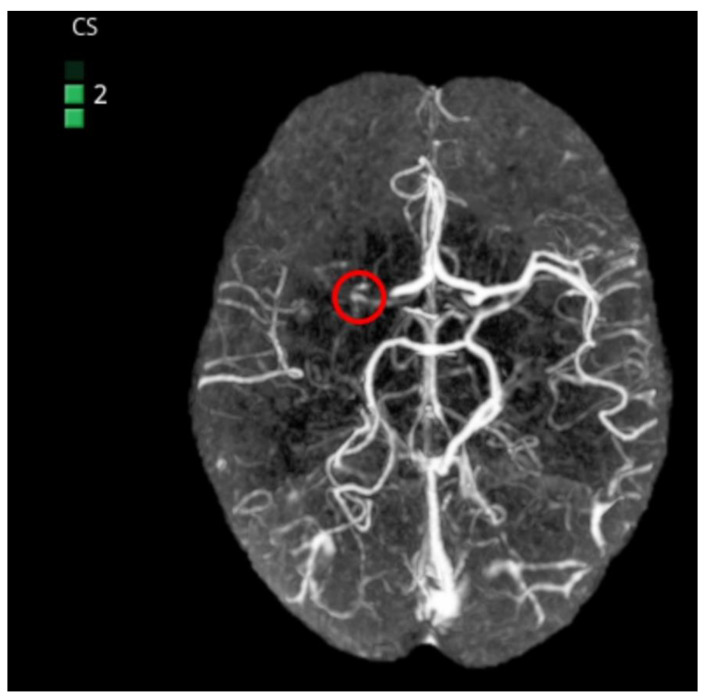
Detection of right middle cerebral artery occlusion by artificial intelligence from CTA. Collateral score = 2.

Automated collateral scoring calculated from computed tomography angiography can identify patients most likely to benefit from treatment. It ranges from 0 (no collaterals) to 3 (complete collaterals). e-CTA provides real-time collateral scoring with the potential to improve consistency of image interpretation and to quantify collateral scores independently (Figure 3).

In the study of 98 patients, 3 experienced neuroradiologists (NRs) independently estimated the CTA-CS, first without and then with knowledge of the e-CTA output, before finally agreeing on a consensus score. Addition of the e-CTA improved the intraclass correlation coefficient (ICC) between NRs from 0.58 (0.46–0.67) to 0.77 (0.66–0.85, *p* = 0.003). Automated e-CTA, without NR input, agreed with the consensus score in 90% of scans with the remaining 10% within one point of the consensus (ICC 0.93, 0.90–0.95). Sensitivity and specificity for identifying favorable collateral flow (collateral score 2–3) were 0.99 (0.93–1.00) and 0.94 (0.70–1.00), respectively. e-CTA correlated with the Alberta Stroke Program Early CT Score (Spearman correlation 0.46, *p* < 0.001), highlighting the value of good collateral flow in maintaining tissue viability prior to reperfusion [61].

In a retrospective review of 77 mechanical thrombectomy patients, collateral scoring (CS) of cerebral arteries using computed tomography angiography (CTA) inversely correlates with the volume of RAPID cerebral blood flow < 30% (*p* < 0.001), RAPID Tmax > 6 s (*p* = 0.011), postintervention stroke volume (*p* < 0.001), and discharge National Institutes of Health Stroke Scale score (*p* = 0.023) [62].

Recently, venous outflow profiles have been investigated in the setting of LVO stroke and were associated with reduced edema formation and good functional outcomes [63].

##### Perfusion Maps

Automatic calculation of perfusion maps and the volume of ischemic core and penumbra are recommended, especially in the problematic case of unknown onset or wake-up stroke. In wake-up stroke patients, evaluating both the DWI-FLAIR and PWI-DWI mismatch patterns will result in the highest yield of thrombolysis treatment [64]. The mismatch between the ischemic core and penumbra is naturally a favorable indication of reperfusion treatment.

Although CT perfusion (CTP) might support decision making, the reliability of CTP is hampered by varying results between different postprocessing software packages. A total of 35 patients with small core volumes in the MR CLEAN trial were analyzed by different methods: IntelliSpace Portal using default settings and with syngo.via using three different settings—default settings, additional smoothing filter, and adjusted settings. The best agreement with RAPID software was provided by syngo.via default settings with additional smoothing (85%) [65]. Compared with RAPID, the Vitrea default setting was noninferior for patients with interventions and superior in penumbra estimation for patients without interventions, as indicated by mean infarct differences and correlations with final infarct volumes [66].

Perfusion maps generated from a temporally sampled helical CTA are another option for infarct core imaging [67,68,69], and color-coded mCTA summation maps may facilitate the easier assessment of acute stroke pathology, including better assessment of collateral status, distal occlusions, carotid pseudo-occlusions, intracranial stenosis, and thrombus permeability [70]. ColorViz may be particularly useful for less experienced readers [71].

**Figure 4:**
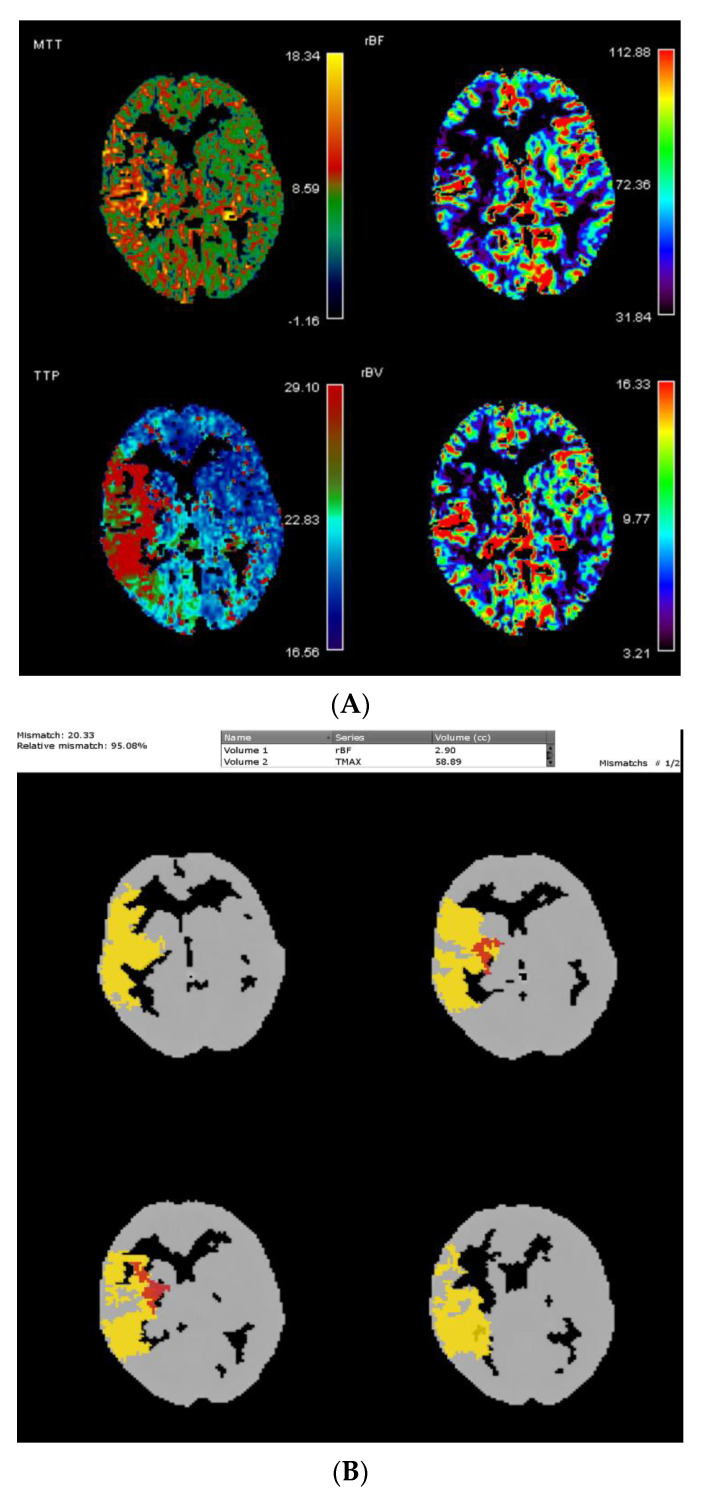
(**A**) Perfusion maps with penumbra in right middle cerebral artery territory and (**B**) relative mismatch = 95.8%.

Recently, neural network-derived perfusion maps were reported to be clinically useful in patients with acute ischemic stroke (Figure 4). It achieved similar performance to standard methods but faster computation time, without relying on an explicit definition of an arterial input function [72]. Such an AI approach to perfusion imaging might thus fasten treatment decisions and dampen the potential human errors associated with the semiautomatic computation of perfusion maps.

##### Differential Diagnosis

When determining the differential diagnosis, the primary goal is to differentiate intracranial hemorrhage from acute ischemia correctly since bleeding is thrombolysis contraindication.

**Figure 5:**
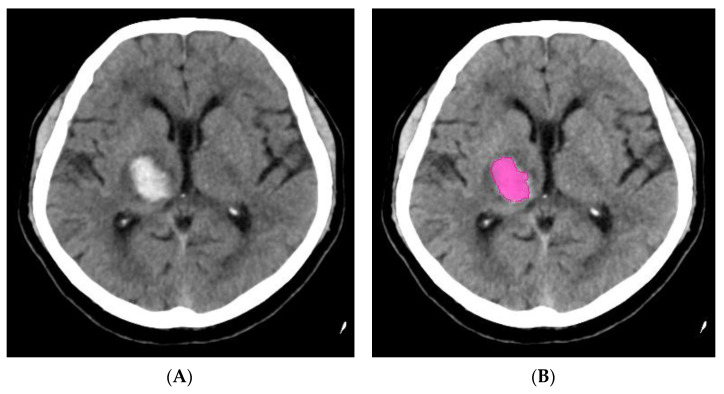
(**A**) NCCT scan—intracerebral hemorrhage and (**B**) intracerebral hemorrhage detected by artificial intelligence.

**Figure 6:**
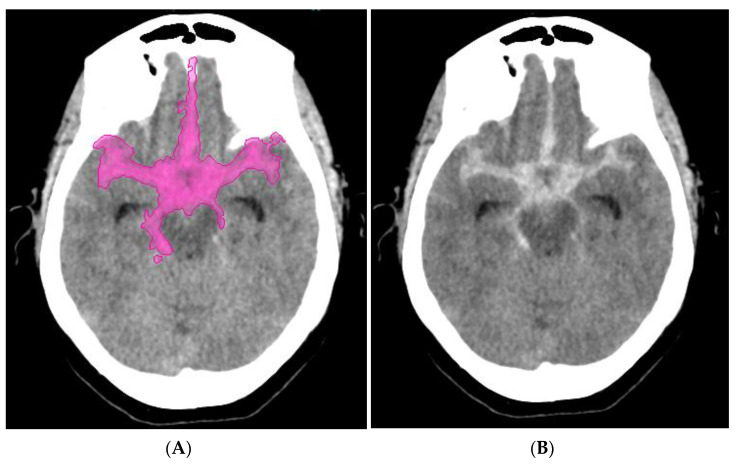
(**A**) NCCT scan—subarachnoid hemorrhage and (**B**) subarachnoid hemorrhage detected by artificial intelligence.

Five subtypes of intracranial hemorrhage (cerebral parenchymal, intraventricular, subdural, epidural, and subarachnoid) can be classified by AI on NCCT (Figure 5 and Figure 6). By using a dataset of only 904 cases for algorithm training, the system achieved a performance level similar to that of expert radiologists in 2 independent test datasets containing 200 cases (sensitivity of 98% and specificity of 95%) and 196 cases (sensitivity of 92% and specificity of 95%) [73].

A novel three-dimensional (3D) joint convolutional and recurrent neural network (CNN-RNN) for the detection of intracranial hemorrhage (ICH) was created based on 2836 subjects (ICH/normal, 1836/1000) with a total of 76,621 slices from NCCT head scans. On average, the algorithm needs less than 30 s for processing to detect and differentiate five subtypes of intracranial hemorrhage (cerebral parenchymal, intraventricular, subdural, epidural, and subarachnoid) in noncontrast head CT.

The algorithm achieved values: ≥ 0.98 for predicting the bleeding and > 0.8 for predicting five subtypes of intracranial hemorrhage. The results were superior to the junior radiology trainees [74].

A big dataset containing a 313.318 head CT scan was created in India for detection of intracranial hemorrhage and its types, fractures of calva, midline shift, and mass effect. A randomly selected part of this dataset (Qure25k dataset) was used for validation, and the rest was used to develop algorithms. An additional validation dataset (CQ500 dataset) was collected in two batches from centers that were different. Areas under the receiver operating characteristic curves (AUCs) were primarily used to assess the algorithms. On the Qure25k dataset, the algorithms achieved an AUC of 0.92 for detecting intracranial hemorrhage (0.90 for intraparenchymal, 0.96 for intraventricular, 0.92 for subdural, 0.93 for extradural, and 0.90 for subarachnoid), 0.92 for fractures, 0.93 for midline shift, and 0.86 for mass effect. On the CQ500 dataset, AUC was 0.94 for intracranial hemorrhage (0.95, 0.93, 0.95, 0.97, and 0.96) 0.96, 0.97, and 0.92, respectively. These algorithms have the potential to automate the triage process [75].

False-positive results may be caused by basal ganglia calcifications [76]. Specific challenges must be overcome to ensure the apparent effectiveness and dependable implementation of these automated tools in a clinical setting [77].

Commercial software Aidoc (Tel Aviv, Israel) was used for analyzing 2011 NCCT scans. Among 373 NCCT scans flagged by the software as positive for acute intracranial hemorrhage, 275 (72.4%) were positive. The accuracy of the intracranial hemorrhage detection was significantly higher for emergency cases than for inpatient cases (96.5% versus 89.4%). AI can help optimize clinical workflow especially in emergency situations [78].

The number of commercially available tools for neuroradiologists increased rapidly. They focused mostly on detection of (1) intracranial hemorrhage detection, (2) stroke imaging, (3) intracranial aneurysm screening, (4) multiple sclerosis imaging, (5) neuro-oncology, (6) head and tumor imaging, and (7) spine imaging [79]. Such tools may play an important role in differential diagnosis soon.

AI additionally includes the incredible potential to transform the standard practice of neuroradiology by progressively improving workflow, diagnosis, and treatment and enhancing the value of quantitative imaging. Modern hospitals and neuroradiologists should care about the direct investments in new AI applications [80].

##### Prognosis

AI can predict the prognosis of acute ischemic stroke treatment, including final ischemic volume [81,82,83], unfavorable clinical outcome [84], good clinical outcomes, as well as hemorrhagic transformation [85]. Knowledge of final ischemic volume may change the treatment strategy. In the case of large volume, treatment can be reconsidered because of an increased chance of hemorrhage and potentially worsening the clinical outcome.

The application of the deep convolution neural networks (CNNs) on predicting final stroke infarct volume using only the source magnetic resonance perfusion images was demonstrated, and the area under the curve of 0.871 ± 0.024 was achieved [81]. Another study concluded that a deep learning model can predict final infarct lesions using baseline magnetic resonance images with a median area under the curve of 0.92 with a volume error of 9 mL [82].

The perfusion–diffusion mismatch model can be outperformed by CNN-based models. Convolutional neural networks-based models achieved higher Dice similarity coefficient (DSC) and area-under-the-curve (AUC) values, compared to those of perfusion–diffusion mismatch models (reperfused patients: AUC = 0.87 ± 0.13 vs. 0.79 ± 0.17, *p* < 0.001; nonreperfused patients: AUC = 0.81 ± 0.13 vs. 0.73 ± 0.14, *p* < 0.01, in CNN vs. perfusion–diffusion mismatch models, respectively) [83].

Baseline e-ASPECTS (commercially available software for the automated ASPECTS) correlated with mRS at 3 months and was predictive of unfavorable outcomes after mechanical thrombectomy. The ASPECTS on baseline CT was scored by e-ASPECTS and three expert raters. Intraclass correlation coefficients between e-ASPECTS and raters were 0.72, 0.74, and 0.76 (all, *p* < 0.001) [84].

Deep learning-based natural language processing may be able to assist with the prediction of the cerebrovascular cause of TIA-like presentations from free-text information. When the convolutional neural network was provided with the history of presenting complaint and magnetic resonance imaging report, AUC (88.3 ± 3.6) was achieved [86].

Source magnetic resonance perfusion-weighted images can be used for the prediction of hemorrhagic transformation severity in acute ischemic stroke patients. Kernel spectral regression performed the best accuracy of 83.7 ± 2.6% from tested methods [85].

Machine learning models were used to predict the clinical follow-up outcome from baseline imaging data and patient information. Among noncontrast CT (NCCT), multiphase computed tomography angiography for regional leptomeningeal score (mCTA-rLMC), and mCTA perfusion lesion visibility (mCTA-arterial and mCTA-venous) techniques, mCTA-venous determined the best accuracy and reliability for detecting early severe ischemia (ASPECTS < 6). It is recommended to include mCTA in imaging protocols in stroke patients with low ASPECTS [68].

Preciseness for the final infarct prediction was studied by comparing the assessment of ischemic changes by expert reading, automated software for NCCT, and CT perfusion on baseline multimodal imaging in another study, which demonstrated high accuracy for the assessment of ischemic changes by different CT modalities with the best accuracy for CBF < 30% and Tmax > 10 s [87].

### 2.5. Inclusion Criteria and Techniques of the Treatment

Endovascular treatment of acute ischemic stroke is satisfactorily established in a limited number of countries. Typical inclusion criteria are defined, but several questions must be explained by research in the future.

Based on the American Heart Association guidelines for the early management of patients with acute ischemic stroke, published in 2019, patients should be treated with means of mechanical thrombectomy if they meet the following criteria [28]:

1. NIHSS score of ≥ 6 (in select cases NIHSS < 6);

2. Prestroke modified ranking scale (mRS) of 0 to 1 (in selected mRS > 1);

3. Age ≥ 18 years old;

4. Causative occlusion of the internal carotid artery or MCA segment 1 (in selected cases, occlusion of M2 segment or M3 segment of the MCA [88];

5. ASPECT of ≥ 6 (in select cases, ASPECT < 6);

6. Treatment initiated within 6 h of symptom onset (in some select cases, up to 24 h: DEFUSE 3 and DAWN trials) [89,90].

Favorable results of studies such as the TENSION study may extend the inclusion criteria in anterior circulation to 1. prolong time window (up to 12 h from stroke symptom onset or from last seen well) and 2. ASPECT score of 3–5 [91].

Endovascular recanalization is performed progressively frequently in the distal cerebral circulation. Medium vessel occlusions (MeVOs) defined as M2/3, A2/3, and P2/3 segment occlusions naturally represent the next frontier [92]. Compared with standard medical treatment with or without intravenous thrombolysis, mechanical thrombectomy for posterior circulation is also a safe and technically feasible treatment option for occlusions of the P2 or P3 segment of the posterior cerebral arteries if performed at comprehensive stroke centers [93].

The benefit of endovascular treatment in posterior circulation is based on the results of the BASILAR study, in which endovascular therapy administered within 24 h of estimated occlusion time is associated with better functional outcomes and reduced mortality in patients with basilar artery occlusion [94]. However, the BEST trial confirmed no evidence of a difference in favorable outcomes of patients receiving endovascular therapy, compared with those receiving standard medical therapy alone, in patients with vertebrobasilar artery occlusion [95].

#### 2.5.1. First-Pass Effect

The first-pass effect is defined as achieving a complete recanalization with a single thrombectomy device pass. There is a significant association between first-pass complete reperfusion and favorable clinical outcome, compared to complete reperfusion after multiple passes [96].

A reperfusion quality is another parameter, which affects clinical outcomes. TICI3 reperfusions are associated with superior outcomes and better safety profiles than TICI2b reperfusions [97].

To increase the recanalization rate, several techniques have been used and its effectivity have been described likewise as usage of balloon guide catheter [98,99,100], BADDASS approach [101], longer stent retrievers [102], correct positioning of stent retrievers [103,104], the push-and-fluff technique [105], novel clot extractor [106,107], Solumbra technique [108], ARTS [109], CAPTIVE [110], SAVE [111], and PROTECT plus [112]. The first-pass effect rate was increased to more than 60%.

Five or more passes of the stent retriever became futile in terms of the recanalization rate and functional outcomes [113]. When mechanical thrombectomy does not retrieve any clot, besides underlying atherosclerotic stenotic lesion, also spontaneous intracranial arterial dissection should be considered as a cause of large vessel occlusion [114]. Dual stent retriever technique [115] and rescue stenting were used as alternatives for failed mechanical thrombectomy [116].

The proper use of intra-arterial adjunctive medications, such as urokinase, tissue-type plasminogen activator (tPA), or glycoprotein IIb/IIIa inhibitors, together with mechanical thrombectomy, may achieve better functional outcomes and lower mortality rates [117].

Using a support vector machine algorithm based on clot radiomics on NCCT, Hofmeister et al. were recently able to predict both first-attempt recanalization with thromboaspiration and the overall number of passages required for successful recanalization. In the future, such AI tools might thus allow a better selection of the first-line endovascular strategy, personalized for each patient based on pretherapeutic imaging [118].

#### 2.5.2. Aspiration vs. Stent Retriever

The aspiration catheters use negative pressure to retrieve the clot without crossing the lesion. Equal functional outcomes of aspiration and stent retriever thrombectomy were observed in unselected patients with anterior circulation infarcts in all occlusion segments. When aspiration was the first-line treatment modality, reperfusion rates were higher and procedure times shorter in all occlusion segments [119].

Noninferiority of aspiration thrombectomy was confirmed by randomized trials Aster [120] and clinically also in Compass [121].

Direct Aspiration First-Pass Technique (ADAPT) is associated with higher rates of functional independence after posterior circulation thrombectomy, compared to stent retriever or combined approach in large “real-world” retrospective study [122]. Meta-analysis suggested that for patients with acute basilar artery occlusion, first-line ADAPT might achieve higher and faster recanalization, comparable neurological improvement, and safety, compared with first-line stent retrievers [123].

Mechanical thrombectomy in distal locations using the new generation of 0.017 microcatheter compatible stent retrievers yields comparable results, compared with ADAPT, in terms of recanalization; however, the use of stent retriever is associated with lower functional independence and higher mortality rate [124].

ADAPT showed similar efficacy to randomized trials using other revascularization techniques in the Promise study [125]. The Aspiration first-pass approach can increase speed and improve recanalization rates and has an impact on dwell time [126,127]. The golden 35 min of stroke intervention with ADAPT affect clinical outcomes [128].

#### 2.5.3. Potential Hemorrhagic Procedural Complications

The catheterization of the occluded artery is potentially associated with the possible risk of artery perforation. This complication is usually very harmful and potentially associated with patient death. To reduce the risk of vessel perforation and subarachnoid hemorrhage sufficiently, the clot can be carefully passed with a wireless microcatheter instead of a microwire [129].

Another source of bleeding can be intracranial aneurysms. Small aneurysms can be undetected prior to occlusion recanalization. All segments of intracranial arteries must be evaluated carefully. Older age hypertensive women are at risk of coincidental aneurysm and stroke with large vessel occlusion as well as the presence of mirror aneurysm. Aspiration thrombectomy is probably a less traumatic technique in such cases [130].

Ruptured hidden aneurysms can be treated by endovascular techniques immediately [130,131], but unruptured ones later, after the patient suffered from acute symptomatic thrombosis, with adequate antiplatelet pretreatment [132].

#### 2.5.4. Neurointerventional Robotics

In the field of neurointervention, robots are clearly on the rise. Diagnostic angiography, carotid artery stenting, and robot-assisted aneurysm coiling were performed successfully [8].

Nowadays, the speed of internet connection can be a limiting factor for robotic systems, which can be controlled and commanded potentially from various places in the world. Additionally, 5G internet network will be helpful for the transfer and collecting of extremely robust databases.

The workstation of robotic systems can be placed in the catheterization laboratory room, and its shielding significantly reduces operator radiation dose. However, such equipment was used successfully in the first-in-human, telerobotic-assisted percutaneous coronary intervention with the operator 20 miles away from the patients. Five patients were included in the study and 53 ms mean delay was recorded by the network [133].

The first-in-human robotic-assisted neuroendovascular intervention was also performed. This was a stent-assisted coiling procedure to treat a large basilar aneurysm and all intracranial steps, including stent placement and coil deployment, were performed with assistance from the robotic system. Such a system has the potential to improve the precision of neuroendovascular procedures while reducing radiation exposure to the interventionalist, and it opens the possibility for stroke treatment. Of course, team training, communication, and preparation are essential to adopt this technology successfully [9].

### 2.6. Intensive Care, Rehabilitation, and Medical Control

AI can be incorporated into intensive-care units for patient monitoring. Due to deep learning, AI improved the prediction of cardiac arrest or acute respiratory failure from 1 to 6 h prior to unset by 40% on average [134].

Stroke leads to motor dysfunction and even permanent disability very often. Rehabilitation training of stroke survivors has become a major social problem. Robots can help patients to carry out reasonable and effective training to improve the motor function of paralyzed extremities. Lower-limb rehabilitation robots can be divided according to training modes: 1. passive mode, 2. active mode, 3. active assist mode, and 4. active resist mode [135]. Several robot types are also available for upper limb training including hand [136].

Task-oriented, task-specific training and virtual reality have been used as an alternative and as an adjunct to conventional rehabilitation [137]. Most advanced human-like robots (e.g., Sophia) with incorporated AI demonstrate incredible potential, especially in natural language conversation training [138].

Patient compliance is crucial for medication effectiveness. Treatment monitoring by mobile phone applications (e.g., WeChat) showed a trend of increasing medication compliance and decreasing ischemic endpoint event rates [139].

## 3. Safety and Ethical Problems

Imaging biobanks would promptly become a necessary infrastructure to organize properly and share the image data from which AI models can be adequately trained. Large biobanks, in addition, offer the potential to simulate disease development and progression [140]. Software developers must maintain moral and ethical behavior and standards. Developed AI needs to be properly certified before clinical use.

Radiology is a prime candidate for the early adoption and implementation of AI [141]. The best practices in data management and personal de-identification are necessary to prevent unacceptable risks of patient reidentification [142,143].

Challenges of data security and patient privacy, along with appropriate consent, remain to be considered [20]. Professionals need to collaborate to eliminate the risk of data hacking attempts as much as possible [144].

Conversely, legal restrictions can inhibit AI research in some parts of the world relative to others, skewing the academic and commercial playing field [145].

AI represents a unique challenge to improve health care by connecting both social and technological infrastructure [20]. AI necessarily becomes part of the educational process of physicians including radiologists [146].

## 4. Discussion

Stroke is one of the leading causes of disability and death. Diagnosis and treatment represent an extraordinarily complex process. Innovative technologies penetrate every step increasingly often. In the prehospital period stroke, symptoms detection remains the crucial key to initiate the process of health care.

Facial asymmetry is one of the most significant symptoms of acute ischemic stroke. The current accuracy to accurately detect facial asymmetry is up to 87% [22]. Unluckily, in the elderly population, a lot of people live separately. A permanent monitoring of the vulnerable people and movement disability detection by sophisticated technologies could represent a key factor to sufficiently reduce the time necessary for the diagnostic process. Without these modern technologies, individuals are dependent on another proper person (usually a neighbor or another family member), who must call emergency. Hopefully, emergency stroke team activation will be performed automatically by innovative technologies soon.

Due to the standardization of initial clinical examination by automated scoring systems and decision-making algorithms, documentation quality can increase up to 100% [26]. The extensive use of telemedicine and teleradiology optimize the consultation and the coordination of the accurate decision for urgently transporting the patient to the endovascular center [28,29,30].

Patient transport is affected by many contributing factors. The most important are the distance to the center, local weather, and traffic situation. Accurate knowledge of real-time traffic information optimizes patient transport, and the precise GPS location of the emergency ambulance allows the stroke team to be ready before the patient’s arrival at the center. In such a case, hospital workflow is blocked for a relatively limited period [31,32].

Patient registration errors can occur instantly for a limited time. QR code-based applications for patient ID information significantly reduce the wrong registration rate [45].

The beneficial effect of time measurement application on treatment decision time reduction (approximately 10 min) was published [37]. Hospital staff naturally work more promptly if a stroke clock timer is visible in the CT scanner room.

Artificial Intelligence has been witnessing monumental growth in bridging the gap between the capabilities of humans and machines. Importantly, 40 to 60% faster scanning is available due to artificial intelligence in magnetic resonance scanners. The only signal is properly selected for image reconstruction while noise is carefully suppressed. Motion artifacts are also suppressed in noncooperative stroke patients.

Brain imaging techniques are required for patient triage. Their primary role is to confirm the diagnosis of acute ischemic stroke and determine core and penumbra volumes and exclusion of diseases that represent a contraindication for treatment. Several AI-based technologies represent new possibilities for how to facilitate processes. Commercially available software with incorporated artificial intelligence can perform ASPECT scoring, artery occlusion detection, collateral score determination, perfusion map calculations, intracranial hemorrhage detection and final infarct prediction, hemorrhagic transformation severity prediction, and patient outcome prediction.

Effectivity of various machine learning algorithms (e.g., linear regression, logistic regression, support vector machines, random forest, deep neural networks) are tested, but convolutional neural networks (CNNs) are commonly used for medical image classification [18].

Three-dimensional (3D) joint convolutional and recurrent neural network (CNN-RNN) constitute a novel method [74]. Convolutional neural networks allow machines to view the world as humans do, perceive it in a similar manner, and use the knowledge for a multitude of tasks.

Enormous shortening of artificial intelligence development can be reached by these methods, which do not require as much time-consuming data preparation and extensive data selection as conventional methods require.

Convolutional neural networks confirmed their capability and superiority in various fields, including ASPECT scoring, artery occlusion detection, perfusion map calculation, and prognostication. Overall, 85% convolutional neural networks sensitivity for ASPECT was published, versus 68% sensitivity of random forest algorithm [55]. Maximum artificial intelligence sensitivity of 97.5% for artery occlusion detection was achieved from NCCT scans [56]. Such sophisticated techniques reduce the risk of patient renal failure and postcontrast nephropathy because contrast media can be reserved for interventional procedures.

Convolutional neural networks calculate perfusion maps faster than standard methods [72]. Convolutional neural networks are capable to detect intracranial hemorrhage with superior results to the junior radiology trainees and sensitivity of 98% [74]. Convolutional neural networks have the capability to predict final infarct volume from baseline magnetic resonance images with AUC > 0.87 and hemorrhagic transformation severity prediction from MR perfusion-weighted images with the best accuracy of 83.7% [81,85].

To increase the recanalization rate, several techniques have been used and its effectivity have been described likewise, including usage of balloon guide catheter [98,99,100], BADDASS approach [101], longer stent retrievers [102], correct positioning of stent retrievers [103,104], the push-and-fluff technique [105], novel clot extractor [106,107], Solumbra technique [108], ARTS [109], CAPTIVE [110], SAVE [111], and PROTECT plus [112]. The first-pass effect rate was increased to more than 60%. Dual stent retriever technique [115] and rescue stenting were used as alternatives for failed mechanical thrombectomy [116].

A higher number of interventional devices are utilized for more complex techniques. Unfortunately, it affects the price of recanalization procedures in a negative way and increasing its unavailability in some countries.

The direct cost of current robotic systems does not allow them to be used more widely worldwide, although their potential is enormous because the operator can efficiently perform interventional procedures from one workplace in different parts of the world.

Most rehabilitation systems focus on the intensive rehabilitation of paretic extremities. Thus far, used basic mechanical rehabilitation systems are being replaced by complex ones, which contain various active sensors. The collected information can be continuously online evaluated, and a rehabilitation program is adapted to the patients’ capabilities. Patients’ independence from another person is crucial for daily life, and it is insufficient rehabilitation, focused on patients’ movement rehabilitation only. Humanoid robots will be optionally used hopefully in the near future to recover patients’ cognitive function [135,136,137,138].

Medication control is important after a patient discharge from the hospital. Modern applications are able to increase patient compliance [139].

## 5. Conclusions

Acute stroke patient health care must be aligned to the concept of “time is brain”.

A permanent monitoring of the vulnerable people and movement disability detection by sophisticated technologies could represent a key factor in the prehospital period to sufficiently reduce the time necessary for the diagnostic process.

The explosion of digital data in recent times allowed the creation of the collection of the quantum of imagining examinations for the development of artificial intelligence for better understanding and interpretation of imagining techniques in acute stroke patients. Owing to artificial intelligence, stroke diagnosis can be conducted very quickly; moreover, artificial intelligence offers an innovative tool for how to predict the final result of endovascular treatment, and in addition, it is a way how to understand this disorder better, improve patient selection who may profit from revascularization, and avoid futile recanalization.

Innovative technologies, artificial intelligence, and robotics are necessary to enhance workflow, because of the lack of human sources, but AI diagnostic methods can also be used as complementary tools to support physicians in image interpretation and decision algorithms.

Endovascular treatment must be focused on how to reasonably achieve first-pass reperfusion. Technical improvement of different devices used for occluded artery recanalization allowed reaching the high rate of the first-pass effect, which is associated with better clinical results.

Humanoid robots will be optionally used to recover patients’ functions. Medication control is important after a patient discharge from the hospital.

## Supplementary Materials

The following are available online at https://www.mdpi.com/article/10.3390/life11060488/s1, Table S1: Role of new technologies and artificial intelligence in acute stroke patient management.
