# Supplementary material for: How to Improve the Management of Acute Ischemic Stroke by Modern Technologies, Artificial Intelligence, and New Treatment Methods
Source: Life (Basel). 2021 May 27;11(6):488. doi: 10.3390/life11060488 (PMC8229281; doi:10.3390/life11060488)
Supplement: Supplementary file 1 [file life-11-00488-s001.zip › life-1206404-supplementary.pdf]

Table S1. Role of new technologies and artificial intelligence in acute stroke patient management.

Time period	Process	New Technology	Tool Example	Impact / Sensitivity of Technology Application	References
Prehospital					
	Stroke symptoms recognition and first aid call				
		Facial asymmetry detection		Current accuracies up to 87%	[22]
	After first aid arrival				
		Standardization of clinical examination	FAST-ED	Improved diagnostic process	[24]
		Automated scoring of patient facial palsy	eFACE	Interclass correlation for eFACE scores: 0.96	[25]
		Decision making algorithms system		Increased documentation quality from 78.66% to 100%	[26]
		Mobile CT		Faster diagnosis	[27]
	Team activation, consultation, and transfer				
		Telemedicine / teleradiology		Improved decision	[28–30]
		Real-time traffic information		Faster patient transport	[31]
		GPS location of the ambulance	STEMI application	Better stroke-team management	[32]
	Prenotification – stroke team activation before patient arrival				
		Workflow application	ESN application	Workflow improvement: door-in-door-out time; 56 ± 34 min vs. 96 ± 45 min, door-to-groin puncture time; 50 ± 7 min vs. 120 ± 25 min, door-to-needle time; 55 ± 12 min vs. 78 ± 16 min (p < 0.0001)	[37]
Hospital					
	Patient registration				
		Patient's ID information application		Reduction of wrong patient registration in hospital from 0.68% to 0.12% (p<0.001)	[45]
	Brain imaging				
		Time measurement application	Stroke Clock app.	Treatment decision time reduction (from 26.00 min to 16.73 min)	[39]
		One-stop management	Flat panel CT performed in operation room	Faster treatment initiation	[40,41]
		CBCT		Posterior fossa artefacts reduction	[42]
		AI	AI in CT scanner	Radiation dose reduction	[43]
		AI	AI in MR scanner	40–60% faster scanning	[43]
		AI	AI in scanner	Faster patient	[47]

	AI	AI in scanner	positioning / examination planning<br>Contrast material doses reduction	[47]
	ASPECT			
	AI		AI non-inferiority compared to neuroradiologists ($p < 0.003$)	[53]
	AI		Greater detection sensitivity with convolutional neural networks algorithms versus random forest learning (85% vs. 68%)	[55]
	Artery occlusion detection			
	AI	AI-occlusion detection on NCCT	Maximum sensitivity of 97.5%	[56]
	AI	AI-occlusion detection on CTA	Sensitivity of 82%	[57]
	Collateral score			
	AI	Collateral score calculated from CTA	Sensitivity for identifying favorable collateral flow (collateral score 2-3) were 0.99	[61]
	Perfusion maps			
	AI	Neural network-derived perfusion maps	Similar performance to standard methods, but faster computation time	[72]
	Differential diagnosis			
	AI	Detection of intracranial hemorrhage on NCCT	Sensitivity 92-98%	[73]
	AI-convolutional and recurrent neural networks	Detection of intracranial hemorrhage on NCCT	Sensitivity 98% - superior results to the junior radiology trainnes	[74]
	Prognosis			
	AI-convolutional and recurrent neural networks	Final infarct prediction form baseline MR images	AUC > 0.87	[81–82]
	AI	Unfavorable outcome prediction	Interclass correlation score > 0.72	[84]
	AI	Hemorrhagic transformation severity prediction form MR PWI	Best accuracy 83.7%	[85]
Techniques of the treatment				
	First pass effect			
	Balloon guide catheter, BADDASS approach, longer stent retrievers, correct positioning of stent retrievers, the		Faster recanalization	[98–112]

		push and fluff technique, novel clot extractor, Solumbra technique, ARTS, CAPTIVE, SAVE, Protect plus.			
Neurointerventional robotics					
		Robot-assisted neurointervention		Tele-robotic-assisted intervention	[8,9,133]
Intensive care, rehabilitation and medical control					
Intensive care monitoring					
		AI	Prediction of cardiac arrest or acute respiratory failure	Improved prediction: 40%	[134]
Hospital / Home					
Rehabilitation					
		Robots		Motor function improvement of paralyzed extremity	[135,136]
		AI	Sofia	Language conversation training	[138]
Home					
Medical control					
		Mobile phone applications	WeChat	Increased medication compliance	[139]
